# Presence of plasmid-mediated quinolone resistance (PMQR) genes in non-typhoidal *Salmonella* strains with reduced susceptibility to fluoroquinolones isolated from human salmonellosis in Gyeonggi-do, South Korea from 2016 to 2019

**DOI:** 10.1186/s13099-021-00431-7

**Published:** 2021-06-01

**Authors:** Sohyun Lee, Nanjoo Park, Sujung Yun, Eunseon Hur, Jiwon Song, Hanna Lee, Yongsug Kim, Sangryeol Ryu

**Affiliations:** 1 Gyeonggi-do Research Institute of Health & Environment, Suwon, 16381 South Korea; 2 Department of Food and Animal Biotechnology, Department of Agricultural Biotechnology, Research Institute for Agriculture and Life Sciences, Center for Food and Bioconvergence, Seoul National University, Seoul, 08826 South Korea

**Keywords:** *Salmonella*, Quinolone resistance, Plasmid-mediated quinolone resistance, PMQR

## Abstract

Non-typhoidal salmonellosis remains a pressing public health problem worldwide. Quinolones, particularly fluoroquinolones, are widely used to treat various infections, including non-typhoidal salmonellosis, which can be a serious illness. The emergence of fluoroquinolone-resistant *Salmonella* has resulted in treatment failure and high mortality rates. In this study, we estimated the presence of plasmid-mediated quinolone resistance (PMQR) genes in *Salmonella enterica* isolated from human salmonellosis patients in South Korea from 2016 to 2019. We evaluated the association of these genes with fluoroquinolone susceptibility. Antimicrobial susceptibility tests for *Salmonella* isolates were performed using the Vitek II system, and the minimum inhibitory concentrations (MIC) of ciprofloxacin and levofloxacin were determined using the E-test method. Plasmid-mediated quinolone resistance (PMQR) genes were detected by PCR amplification and quinolone resistance-determining regions (QRDRs) of the *gyrA* and *parC* genes were analyzed following Sanger sequencing of the PCR products. Thirty-four *Salmonella* strains with reduced susceptibility to fluoroquinolones (ciprofloxacin MIC ≥ 0.125 µg/mL and levofloxacin MIC ≥ 0.25 µg/mL) were selected from 208 human clinical *Salmonella* isolates. Among them, 22 *Salmonella* strains harbored one PMQR gene (*qnrA*, *qnrB*, or *qnrS*), and three *Salmonella* strains carried two PMQR genes (*qnrS* and *aac(6′)-Ib-cr* or *qnrA* and *qnrB*). *qnrS* was the most common PMQR gene. Serotyping revealed that *Salmonella* 4,[5]12:i:- (32.4%, 11/34) and *Salmonella* Typhimurium (29.4%, 10/34) were the two most predominant serovars, and Multi-locus sequence typing (MLST) showed that ST19 and ST34 were the most frequent sequence types. In conclusion, *qnr* gene-positive *Salmonella* 4,[5],12:i:- and *Salmonella* Typhimurium were the main serovars responsible for reduced susceptibility to fluoroquinolones. Therefore, our findings suggest that PMQR-positive *Salmonella* strains, which can be isolated from various samples including human, food, and the environment, should be carefully monitored.

**Supplementary Information:**

The online version contains supplementary material available at 10.1186/s13099-021-00431-7.

## Background

Salmonellosis is a disease caused by *Salmonella* that usually produces acute onset of fever, abdominal pain, diarrhea, and vomiting. Antimicrobial therapy is not recommended in healthy individuals with mild or moderate infection [1]. This is due to the use of antimicrobials may not shorten the duration of clinical symptoms, but is rather a risk for prolonged *Salmonella* infection [2]. However, high-risk groups including infants, the elderly, and immunocompromised patients may require antimicrobial therapy [1]. Quinolones, particularly fluoroquinolones (e.g., ciprofloxacin and levofloxacin), are a critically important antimicrobial class, and invasive *Salmonella* infections in adults are commonly treated with quinolones. However, because quinolones are frequently used in human and veterinary medicine, resistance to this antimicrobial class has evolved among *Salmonella* strains [3]. Fluoroquinolone resistance is mostly associated with chromosomal mutations in the bacterial genes encoding targeted enzymes, DNA gyrase and topoisomerase IV (quinolone resistance determining region, QRDR). However, fluoroquinolone resistance can also be acquired by plasmid encoded genes (plasmid-mediated quinolone resistance, PMQR). There are several well-known PMQR gene groups, including *qnr* families (*qnrA*, *qnrB*, *qnrC*, *qnrD*, *qnrE*, *qnrS*, and *qnrVC*), antibiotic efflux pump-coding genes (*qepA* and *oqxAB*), antibiotic modification enzyme gene (*aac(6)Ib-cr*), and a newly described phosphorylase gene (*crpP*) [4]. According to previous studies, the *qnr*, *aac(6′)-Ib-cr*, and *qepA* genes are commonly detected in South Korea; therefore, we chose these genes to investigate in this study [5, 6]. The Qnr is a pentapeptide repeat protein that protects DNA gyrase and topoisomerase IV by inhibiting quinolone [7, 8]. The *aac(6′)-Ib-cr* gene encodes aminoglycoside acetyltransferase that simultaneously induces resistance against aminoglycoside and fluoroquinolone [9]. PMQR facilitates the spread of quinolone resistance, leading to the emergence of high quinolone resistance, making infections difficult to treat [10]. Therefore, the presence of PMQR genes in *Salmonella* strains with reduced susceptibility to fluoroquinolones indicates that continuous monitoring and clinical attention are required. Although PMQR genes confer reduced susceptibility of bacteria to fluoroquinolones, their influence on nalidixic acid susceptibility is minor [11]. The United States National Antimicrobial Resistance Monitoring System (NARMS) has indicated the presence of PMQR genes among *Salmonella* and other enteric bacteria isolated from humans, retail meat, and food animals in the United States. Additionally, NARMS recently reported an increase in the proportion of ciprofloxacin-non-susceptible strains lacking nalidixic acid resistance [12]. In Canada, a relatively high prevalence of PMQR genes has been reported in human isolates of non-typhoidal *Salmonella* with resistance and reduced susceptibility to fluoroquinolones [13]. Likewise, PMQR gene-positive *Salmonella* 4,[5],12:i:- were recently isolated from pigs, chickens, humans, geese, and cats in China [14]. In this study, we aim to estimate the presence of the plasmid-mediated quinolone resistance genes and their association with fluoroquinolone susceptibility in non-typhoidal *Salmonella* isolates from human clinical samples in South Korea from 2016 to 2019.

## Methods

Thirty-four nontyphoidal *Salmonella* strains with intermediate resistance to quinolone or fluoroquinolone were selected and evaluated from 208 human clinical *Salmonella* strains. The strains were isolated from fecal samples of diarrhea patients in Gyeonggi-do, South Korea, by the Research Institute of Health & Environment from 2016 to 2019 (see Additional file 1). The rectal swab samples were plated on *Salmonella*–*Shigella* (SS) agar (Oxoid, Basingstoke, UK) and incubated at 37 °C for 18 to 24 h. Isolates with typical *Salmonella* phenotypes were confirmed using the Vitek II system with a GN card (bioMerieux Inc., Marcy l’Etoile, France). *Salmonella* serotyping was done according to the White–Kauffmann–Le Minor scheme using slide agglutination test (O antigen) and tube agglutination test (H antigen) with antisera. The isolates were serotyped using the somatic (O) (provided from Korea Disease Control and Prevention Agency, KDCA) and flagella (H) antisera (Difco, Detroit, MI, USA). The absence of *hin* gene in the monophasic variant of *Salmonella* Typhimurium was confirmed by PCR. Antimicrobial susceptibility tests were performed using the Vitek II system with the AST-N169 card (bioMerieux Inc.) according to the manufacturer’s instructions. The minimum inhibitory concentrations (MIC) of ciprofloxacin and levofloxacin were determined using the E-test method (bioMerieux Inc.). Quinolone and fluoroquinolone MIC values were confirmed according to CLSI guidelines [15]. In *Salmonella*, a ciprofloxacin MIC of 0.12–0.5 µg/mL is defined as intermediate and MIC ≥ 1 µg/mL is defined as resistant, while a levofloxacin MIC of 0.25–1 µg/mL is defined as intermediate and MIC ≥ 2 µg/mL is defined as resistant. Additionally, a nalidixic acid MIC ≥ 32 µg/mL is defined as resistant, and there is no intermediate category. Total DNA was extracted from overnight cultures of *Salmonella* isolates using the Nextractor NX-48 system and NX-48 bacterial DNA kits (Genolution Inc., Seoul, Korea). PMQR genes (*qnrA*, *qnrB*, *qnrS*, *aac(6′)-Ib-cr*, and *qepA*) were detected by PCR amplification using primers described in previous studies [16–18]. The QRDR region of the *gyrA* and *parC* genes was each PCR-amplified using previously described primers [19]. PCR products were purified and Sanger sequenced by Macrogen Inc, Korea. The nucleotide sequences of QRDRs in *gyrA* and *parC* genes were compared with the counterpart sequences of quinolone-susceptible reference strain *Salmonella* Typhimurium LT2 (GenBank Accession number AE006468) using BLAST. Seven housekeeping genes (*aroC*, *dnaN*, *hemD*, *hisD*, *purE*, *sucA*, and *thrA*) were amplified using previously reported MLST primers (see Additional file 2). PCR products were sequenced by Macrogen (South Korea). Each isolate’s sequence type (ST) was assigned according to the PubMLST website. Phylogenetic analyses of the isolates using MLST-based clusters were conducted with BioNumerics software (Applied Maths, Sint-Martens-Latem, Belgium). The correlation between PMQR genes and MIC values was analyzed by Fisher’s exact test using GraphPad Prism software v.5 (GraphPad Software Inc., La Jolla, CA). A P-value < 0.05 was considered to indicate statistical significance.

## Results and discussion

Thirty-four *Salmonella* strains with reduced susceptibility to fluoroquinolones were identified from Gyeonggi-do, South Korea, from 2016 to 2019. *Salmonella* 4,[5],12:i:- (32.4%, 11/34) and *Salmonella* Typhimurium (29.4%, 10/34) were the predominant serovars. Isolated *Salmonella* serovars showing intermediate resistance to quinolones, particularly fluoroquinolones, were presented in Table 1. In the last two decades, *Salmonella* 4,[5],12:i:- has rapidly emerged worldwide [14]. In South Korea, the first foodborne outbreak of *Salmonella* 4,[5],12:i:- was reported in 2008 [20]; the same serovar, which exhibits nalidixic acid resistance, was isolated from pigs and chickens in South Korea [21]. Similarly, most *Salmonella* 4,[5],12:i:- isolates from chickens, geese, and cats in China were resistant to nalidixic acid (52.5%) [14]. Isolates of enrofloxacin-resistant *Salmonella* 4,[5],12:i:- from swine in the United States have also been reported [22].

**Table 1:** Isolation and antibiotic resistance information for Salmonella isolates evaluated in this study

#	Serotype	Year	ST	AST^a^.	QRDR	PMQR	MIC^b^.	
				Nalidixic acid			Ciprofloxacin	Levofloxacin
29	Typhimurium	2016	19	R	GyrA(D87Y)	–	0.125	0.25
43	Typhimurium	2017	36	S	–	*qnrS1*	0.125	0.38
44	Typhimurium	2017	36	S	–	*qnrS1*	0.125	0.38
48	Typhimurium	2017	19	S	–	*qnrS1*	0.125	0.25
63	Typhimurium	2017	19	R	GyrA(D87Y)	–	0.125	0.25
53	Typhimurium	2018	19	R	–	*qnrS1*	0.19	0.38
17	Typhimurium	2018	16	R	GyrA(S83F)	–	0.125	0.25
19	Typhimurium	2018	19	R	GyrA(D87Y)	–	0.125	0.25
20	Typhimurium	2018	19	R	–	*qnrS1*	0.25	0.5
21	Typhimurium	2018	19	R	GyrA(D87Y)	*qnrS1*	0.25	0.75
13	I 4,[5],12:i:-	2017	34	R	–	*qnrS1*	0.19	0.38
22	I 4,[5],12:i:-	2018	34	S	–	*qnrS1*	0.125	0.25
23	I 4,[5],12:i:-	2018	34	R	–	*qnrS1*	0.19	0.38
24	I 4,[5],12:i:-	2018	19	S	–	*qnrS1*	0.125	0.38
25	I 4,[5],12:i:-	2018	34	S	–	*qnrS1*	0.125	0.25
54	I 4,[5],12:i:-	2018	34	R	–	*qnrS1*	0.19	0.5
55	I 4,[5],12:i:-	2018	34	R	–	*qnrS1*	0.19	0.38
57	I 4,[5],12:i:-	2018	34	R	–	*qnrS1*	0.125	0.38
59	I 4,[5],12:i:-	2018	34	R	–	*qnrS1*	0.19	0.5
50	I 4,[5],12:i:-	2018	19	R	–	*qnrS1*	0.25	0.5
61	I 4,[5],12:i:-	2019	34	R	–	*qnrS1*	0.25	1
11	Rissen	2017	469	R	GyrA(S83Y), ParC(T57S)	–	0.125	0.5
12	Rissen	2017	469	R	GyrA(S83Y), ParC(T57S)	–	0.125	0.38
2	Saintpaul	2017	27	R	–	*qnrS1, aac(6’)-Ib-cr*	0.5	0.38
3	Saintpaul	2017	27	R	–	*qnrS1, aac(6’)-Ib-cr*	0.5	0.38
8	Enteritidis	2017	11	R	GyrA(D87N)	–	0.032	0.25
10	Kentucky	2017	11	R	GyrA(D87G)	–	0.047	0.25
15	Carno	2017	1992	R	ParC (T57S)	*qnrA, qnrB*	0.19	0.5
16	Agona	2017	13	R	ParC (T57S)	*qnrB*	0.125	0.5
18	Hato	2018	13	R	ParC (T57S)	*qnrS1*	0.125	0.5
32	Duesseldorf	2016	292	R	GyrA(S83F), ParC(T57S)	–	0.125	0.25
51	Braenderup	2018	311	R	ParC(T57S)	*qnrS2*	0.19	0.5
52	Derby	2018	Undetermined	S	–	*qnrS1*	0.125	0.38
62	Newport	2019	214	R	–	*qnrS1*	0.125	0.25

*ST* sequence typing,* QRDR* quinolone-resistance determining region,* PMQR* plasmid-mediated quinolone resistance,* MIC* minimum inhibitory concentration

^a^Vitek II system with AST-N169 card

^b^E-test method

All 34 isolates (100%) showed reduced susceptibility to levofloxacin, 32 isolates (94.1%) showed reduced susceptibility to ciprofloxacin, and 27 isolates (79.4%) were also resistant to nalidixic acid. We obtained seven non-typhoidal *Salmonella* isolates that showed reduced susceptibility to fluoroquinolones and susceptibility to nalidixic acid. Resistance to nalidixic acid could be related to reduced susceptibility to fluoroquinolones because it typically required chromosomal mutations in the quinolone resistance-determining region (QRDR) or acquisitions of PMQR genes (Table 1). Reduced susceptibility to fluoroquinolones without nalidixic acid resistance indicated PMQR presence [10], and the US NARMS has found higher percentages of isolates with reduced susceptibility to ciprofloxacin than nalidixic acid resistance since 2005 [12].

PMQR genes were detected in 25 (73.5%) out of 34 *Salmonella* isolates, including one (2.9%) isolate positive for *qnrA*, two (5.9%) isolates positive for *qnrB*, 23 (67.6 %) positive for *qnrS*, and two (5.9 %) positive for *acc(6′)-Ib-cr* (Table 1). The correlation between PMQR genes and MIC values was statistically insignificant. According to previous studies, the *qnr* gene family was predominantly detected in *Salmonella* strains isolated from travelers. Most of the strains carrying the *qnrS* gene showed reduced susceptibility to ciprofloxacin [21]. The *qnrS* gene was mostly harbored by *Salmonella* 4,[5],12:i:- and *Salmonella* Typhimurium [23, 24]. The *qepA* gene was not detected in any isolate. According to a study on PMQR gene prevalence in clinical *Enterobacteriaceae* isolates from South Korea, *qnrB* was the most frequently observed PMQR gene before 2000, whereas *qnrS*, *aac(6′)-Ib-cr*, and *qepA* emerged after 2000 [25]. Although most PMQR-positive isolates possessed only one gene, two *Salmonella* Saintpaul isolates were positive for *qnrS* and *acc(6′)-Ib-cr* and one *Salmonella* Carno isolate was positive for *qnrA* and *qnrB*. Among the PMQR gene-positive *Salmonella* isolates from patients and turkey meat, *Salmonella* Saintpaul carrying one *qnrS1* gene was reported in the Netherlands and Denmark [26, 27]. *Salmonella* Carno is a rare serotype, and this serovar has not been extensively studied.

**Fig. 1:**
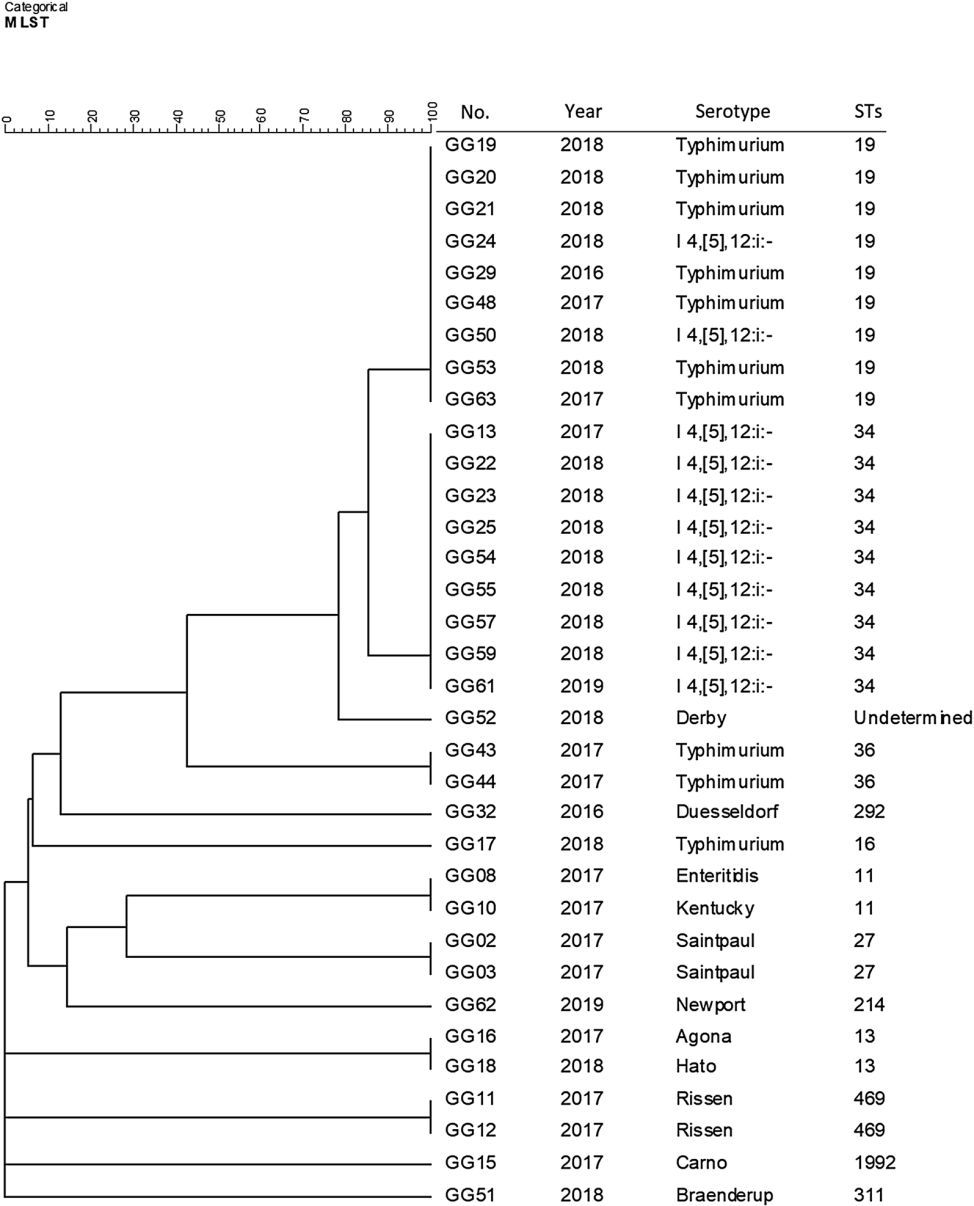
Phylogenetic tree based on MLST sequence typing of PMQR-positive *Salmonella* isolates from South Korea. The cluster analysis was performed using the categorical coefficient and the UPGMA in BioNumerics

To estimate genetic correlations, multi-locus sequence typing (MLST) was conducted. Nine isolates of ST19 type (seven *Salmonella* Typhimurium and two *Salmonella* 4,[5],12:i:-), 9 isolates of ST34 type (nine *Salmonella* 4,[5],12:i:-), 2 isolates of ST36 type (two *Salmonella* Typhimurium), ST27 type (two *Salmonella* Saintpaul), ST13 type (one *Salmonella* Agona and one *Salmonella* Hato) and ST469 type (two *Salmonella* Rissen) were identified. ST19 and ST34 were the predominant STs in *Salmonella* Typhimurium and *Salmonella* 4,[5],12:i:-, which had high genetic diversity but were over 80% similar to each other (Fig. 1). ST19 and ST34 are more common in *Salmonella* Typhimurium STs responsible for infections worldwide [28, 29]. ST19 and ST34 prevalence in *Salmonella* 4,[5],12:i:- has significantly increased in Canada, and some of these STs demonstrate quinolone resistance [30]. It has been reported that ST19 is significantly associated with ciprofloxacin resistance in China [31] and that ST34 is linked to the nalidixic acid resistance in Africa [32]. *Salmonella* Agona ST13 was the most prevalent serovar isolated from chicken meat in Sri Lanka [33], and a *Salmonella* Agona strain isolated from chicken meat in China possessed a T57S substitution in ParC and carried *qnrS* [34]. *Salmonella* Rissen ST469 was isolated from ready-to-eat mussels in Spain and pork products in Portugal [35, 36]. Moreover, ST469 was the third most predominant ST isolated from pork samples in China and nearly one-third of the ST469 isolates were resistant to ciprofloxacin [37]. However, the sequence type of one *Salmonella* Derby isolate could not be determined by MLST, suggesting the possibility that a potential novel ST of *Salmonella* Derby has emerged in South Korea.

## Conclusions

We isolated 34 *Salmonella* strains with reduced susceptibility to fluoroquinolones from human salmonellosis. Among them, *Salmonella* 4,[5],12:i:- and *Salmonella* Typhimurium were the most common serovars, and MLST revealed that ST19 and ST34 were the predominant lineages, with a high genetic similarity of over 80%. The spread of plasmid-mediated antibiotic resistance in ST19 and ST34 strains requires careful attention in South Korea. Furthermore, all isolates carried one or two of the PMQR genes, suggesting that various genes associated with quinolone resistance can be transferred horizontally among *Enterobacteriaceae*, causing human infections in South Korea.

## Supplementary Information

**Additional file 1: Table S1.** *Samonella* serovars isolated from clinical samples in Gyeonggi-do, South Korea.

**Additional file 2: Table S2.** Primer sequences used for this experiment.

## Data Availability

Data sharing not applicable to this article.
