# Supplementary material for: Presence of plasmid-mediated quinolone resistance (PMQR) genes in non-typhoidal Salmonella strains with reduced susceptibility to fluoroquinolones isolated from human salmonellosis in Gyeonggi-do, South Korea from 2016 to 2019
Source: Gut Pathog. 2021 Jun 1;13:35. doi: 10.1186/s13099-021-00431-7 (PMC8167944; doi:10.1186/s13099-021-00431-7)
Supplement: Supplementary file 1 — Additional file 1: Table S1. Samonella serovars isolated from clinical samples in Gyeonggi-do, South Korea. [file 13099_2021_431_MOESM1_ESM.docx]

**Table S1. *Samonella* serovars isolated from clinical samples in Gyeonggi-do, South Korea**

| Serovars | Number of strains |
| --- | --- |
| I 4,[5],12:i:- | 57 |
| Typhimurium | 33 |
| Bareilly | 13 |
| Stanley | 11 |
| Thompson | 11 |
| Infantis | 10 |
| Enteritidis | 9 |
| Agona | 8 |
| Braenderup | 6 |
| Schwarzengrund | 4 |
| Paratyphi A | 3 |
| Poona | 3 |
| Rissen | 3 |
| Saintpaul | 3 |
| Sandiego | 3 |
| Schleissheim | 3 |
| Bardo | 2 |
| Duesseldorf | 2 |
| Livingstone | 2 |
| Newport | 2 |
| Othmarschen | 2 |
| Paratyphi B | 2 |
| Reading | 2 |
| Amager var. 15^+^ | 1 |
| Anatum | 1 |
| Carno | 1 |
| Derby | 1 |
| Dessau | 1 |
| Haifa | 1 |
| Hato | 1 |
| Houston | 1 |
| Kentucky | 1 |
| Mbandaka | 1 |
| Nitra | 1 |
| Surat | 1 |
| Teddington | 1 |
| Urbana | 1 |
| Total | 208 |
