# Supplementary material for: Presence of plasmid-mediated quinolone resistance (PMQR) genes in non-typhoidal Salmonella strains with reduced susceptibility to fluoroquinolones isolated from human salmonellosis in Gyeonggi-do, South Korea from 2016 to 2019
Source: Gut Pathog. 2021 Jun 1;13:35. doi: 10.1186/s13099-021-00431-7 (PMC8167944; doi:10.1186/s13099-021-00431-7)
Supplement: Supplementary file 2 — Additional file 2: Table S2. Primer sequences used for this experiment. [file 13099_2021_431_MOESM2_ESM.docx]

**Table S2. Primer sequences used for this experiment**

| PMQR Primer | Sequence (5' to 3') | size (bp) |
| --- | --- | --- |
| qnrA F | ATTTCTCACGCCAGGATTTG | 516 |
| qnrA R | GATCGGCAAAGGTTAGGTCA |  |
| qnrB F | GATCGTGAAAGCCAGAAAGG | 469 |
| qnrB R | ACGATGCCTGGTAGTTGTCC |  |
| qnrS F | ACGACATTCGTCAACTGCAA | 417 |
| qnrS R | TAAATTGGCACCCTGTAGGC |  |
| aac(6')-Ib-cr F | TTGCGATGCTCTATGAGTGGCTA | 482 |
| aac(6')-Ib-cr R | CTCGAATGCCTGGCGTGTTT |  |
| qepA F | GGACATCTACGGCTTCTTCG | 720 |
| qepA R | AGCTGCAGGTACTGCGTCAT |  |

| QRDR Primer | Sequence (5' to 3') | size (bp) |
| --- | --- | --- |
| STGYRA1 | TGTCCGAGATGGCCTGAAGC | 450 |
| STGYRA12 | CGTTGATGACTTCCGTCAG |  |
| STPARC1 | ATGAGCGATATGGCAGAGCG | 400 |
| STPARC2 | TGACCGAGTTCGCTTAACAG |  |

| MLST Primer | Sequence^a.^ (5' to 3') | size (bp) |
| --- | --- | --- |
| aroC F | CCTGGCACCTCGCGCTATAC | 826 |
| aroC R | CCACACACGGATCGTGGCG |  |
| dnaN F | ATGAAATTTACCGTTGAACGTGA | 833 |
| dnaN R | AATTTCTCATTCGAGAGGATTGC |  |
| hemD F | ATGAGTATTCTGATCACCCG | 666 |
| hemD R | ATCAGCGACCTTAATATCTTGCCA |  |
| hisD F | GAAACGTTCCATTCCGCGCAGAC | 894 |
| hisD R | CTGAACGGTCATCCGTTTCTG |  |
| thrA F | GTCACGGTGATCGATCCGGT | 852 |
| thrA R | CACGATATTGATATTAGCCCG |  |
| sucA F | AGCACCGAAGAGAAACGCTG | 643 |
| sucA R | GGTTGTTGATAACGATACGTAC |  |
| purE F | ATGTCTTCCCGCAATAATCC | 510 |
| purE R | TCATAGCGTCCCCCGCGGATC |  |

a. Available from:

https://enterobase.readthedocs.io/en/latest/mlst/mlst-legacy-info.html (accessed on 27 January 2021)
